# The incidence, prevalence, and survival of systemic sclerosis in the UK Clinical Practice Research Datalink

**DOI:** 10.1007/s10067-018-4182-3

**Published:** 2018-06-30

**Authors:** Jeremy G. Royle, Peter C. Lanyon, Matthew J. Grainge, Abhishek Abhishek, Fiona A. Pearce

**Affiliations:** 1 Department of Rheumatology, Nottingham University Hospitals NHS Trust, Nottingham, UK; 2 Division of Epidemiology and Public Health, University of Nottingham, Nottingham, UK; 3 Division of Academic Rheumatology, University of Nottingham, Nottingham, UK

**Keywords:** Systemic sclerosis, Epidemiology, Incidence, Prevalence, Mortality

## Abstract

To estimate the incidence, prevalence, and survival of systemic sclerosis in the United Kingdom. We conducted a historical cohort study using data from the Clinical Practice Research Datalink (CPRD). We calculated the incidence and survival of systemic sclerosis between 1994 and 2013 and examined its association with age, sex, and socioeconomic status. We calculated point prevalence on 1 July 2013 and examined its association with the same exposures. We identified 1327 cases with incident systemic sclerosis. Annual incidence was 19.4 per million person-years between 1994 and 2013. The incidence was 4.7 times higher in women than in men, was not influenced by socioeconomic status, and has remained stable over the 20 year study period. The peak age of onset was 55–69 years. Survival at 1, 5, and 10 years was 94.2, 80.0, and 65.7%, respectively. The prevalence was 307 (290–323) per million with the highest prevalence in the 70–84 years age group. We estimate there are currently 1180 new cases of systemic sclerosis each year in the UK, and 19,390 people living with systemic sclerosis. Due to the predicted growth and aging of the population, we predict a 24% increase in incident cases and 26% increase in prevalent cases in 20 years’ time. Our estimates of incidence and prevalence are higher than previously reported in the UK, but similar to recent USA and Swedish studies, and do not support a north-south gradient of the occurrence of systemic sclerosis in Europe.

## Introduction

Systemic sclerosis is a rare autoimmune disease of unknown etiology characterized by skin fibrosis and internal organ involvement. The incidence and prevalence of systemic sclerosis have been reported to vary widely with incidence estimated between 4 and 43/million person years [1–3], and prevalence between 88 and 443/million [4, 5]. It seems the incidence and prevalence may be influenced by race [6, 7], but whether there are true geographical differences in occurrence in Caucasian populations is less clear.

The literature before 2006 is summarized in a systematic review [8], which proposed a North-South gradient in Europe with lower rates in Northern European countries (UK, Finland, and Iceland [1, 2, 5, 9]) compared to Southern European ones (France and Greece [7, 10]). Studies published since this have continued to report high incidences in Southern Europe (Spain, Croatia and Italy) [3, 11, 12] but contradictory rates in Northern Europe with low annual incidence of 6–11 per million in Norway [13] and a higher rate 19/million person-years in Southern Sweden [14]. Incidence and prevalence in the USA, Canada, and Australia are reported at the higher end of this range [4, 15, 16].

Previous studies of the epidemiology of systemic sclerosis in the UK were small [1, 5, 17], and the epidemiology of systemic sclerosis has never been examined in a nationwide population-based study, which reduces the biases that are inherent in smaller hospital-based studies. The availability of Clinical Practice Research Datalink (CPRD) (a longitudinal database of consultation based patient records from UK general practice) gives us an opportunity to examine the epidemiology of systemic sclerosis in a large population that is representative of the UK population [18].

Understanding the incidence and prevalence of systemic sclerosis will help to address the healthcare needs and aid service planning for this rare disease, both now and in the future. Such service planning may include the setting up, staffing, and resourcing of specialist treatment centers. It may also add to the debate about whether occurrence of systemic sclerosis is lower in Northern Europe.

The aim of this study was to estimate the incidence, prevalence, and mortality rates of systemic sclerosis in the UK and to explore temporal trends in its incidence. We also investigated the effects of age, sex, and socioeconomic status on incidence, prevalence, and mortality.

## Patients and methods

### Study design and population

The UK healthcare system is well-suited to capturing diseases which are managed both in hospitals and in the community. In the UK, everybody is registered with a general practitioner (GP) who co-ordinates their healthcare including referrals to secondary care. For example, when a patient is discharged from hospital, or attends a hospital clinic, a letter is written to inform the GP of any new diagnoses, and these are added to the GP record. By using GP data, we would expect to capture people with the full spectrum of scleroderma, from the mildest to the most severe, and would not expect to need to interrogate hospital databases or discharge letters from hospital to identify additional cases.

This is a historical cohort study containing all 684 general practices contributing data to the CPRD in 2015. The CPRD is a longitudinal general practice database of approximately 13 million people who have contributed data since 1987, and approximately 6% of the UK population are currently contributing data [19]. The database contains general practices from all four countries in the UK and includes information on demographics, diagnoses, referrals, medications, and tests. It is deemed to be representative of the UK population [18, 20]. We followed the CPRD’s recommendations for selecting research quality patient records and periods of quality data recording by including people contributing “acceptable” quality data in “up to standard” practices. Our study was conducted between 1 January 1994 and 31 December 2013.

### Case definition

We compiled lists of Read codes for a diagnosis of systemic sclerosis by searching the description fields of the Read code dictionary and excluding irrelevant codes, using a method described by Dave and Petersen [21]. Synonyms searched for were systemic sclerosis, scleroderma, and CREST. Only Read codes that were specific for a diagnosis of systemic sclerosis were used, and codes for localized scleroderma were excluded. Read codes are available as an online supplement and at clinicalcodes.org [22]. We did not validate the diagnosis of systemic sclerosis externally, because (1) validation of other chronic autoimmune diseases in the CPRD has shown positive predictive values of > 90% [23, 24], and (2) GPs would be unlikely to give a patient a Read code for systemic sclerosis unless it had been confirmed by a hospital specialist [25]. Incident cases were defined as people with a first record of a Read code during the study period, and prevalent cases were people who had ever had a code for systemic sclerosis. We only included incident cases with a least 1 year of disease-free follow up in the CPRD prior to their diagnosis in order to reduce the chance of prevalent cases being misclassified as incident cases [26].

### Data sources

All data were extracted from the CPRD files except for the 2010 English Index of Multiple Deprivation (IMD10) which was used as a proxy for socioeconomic status. IMD10 was supplied, via a linkage agreement, by the Office for National Statistics (ONS) and was available for patients in English practices that had consented to participate in the linkage scheme (~ 60%) [27].

### Statistical analysis

We categorized age into groups (0–15, 16–39, 40–54, 55–69, 70–84, and 85+ years) and used IMD-10 quintiles, where quintile 1 is the most deprived, and quintile 5 the least deprived. A further “missing” IMD category was created to maintain all participants in the analyses.

We calculated crude incidence rates, and stratified these by age group, sex, year of diagnosis, and IMD-10 quintile. The denominator was all people contributing acceptable quality data in up to standard practices to the CPRD during the study period. Unadjusted incidence rate ratios were obtained by fitting variables individually in separate Poisson regression models. Mutually adjusted incidence rate ratios were obtained by fitting age group, sex, and IMD-10 quintiles as a priori confounders in a single Poisson regression model.

We calculated point prevalence per million people on 1 July 2013 and stratified this in the same way as for incidence. To calculate this, we divided the number of people with a Read code for systemic sclerosis who were alive and contributing data on this date by the total number of people alive and contributing data on this date. We used logistic regression models to calculate odds ratios (ORs) and 95% confidence intervals (CIs), which provides a good estimation of the relative risk for rare outcomes such as this. Unadjusted ORs were obtained by fitting variables individually in separate logistic regression models. Mutually adjusted ORs were obtained by fitting age group, sex, and IMD-10 quintiles as a priori confounders in a single logistic regression model.

Kaplan-Meier methods were used to estimate survival at 1, 5, and 10 years after diagnosis. We used Cox regression to estimate hazard ratios (HRs) for the effect of sex, age group, and IMD-10 quintile on mortality. Unadjusted HRs were obtained by fitting variables individually in separate Cox regression models. Mutually adjusted HRs were obtained by fitting age group, sex, and IMD-10 quintiles as a priori confounders in a single Cox regression model.

We used direct standardization of the age-specific incidence rates to the ONS age-stratified UK population now and projected UK population in 20 years, to estimate the number of expected incident and prevalent cases in the UK now and in 2037, assuming that incidence will not change over the next 20 years.

All analyses were performed using Stata 14 statistical software (Statacorp, Texas, USA).

### Ethics

Independent Scientific Advisory Committee (ISAC) for MHRA Database Research approval was obtained for this study on October 26, 2016 (protocol 16_190R).

### Reporting guidelines

This study has been reported following the RECORD guidelines for reporting of studies conducted using observational routine-collected health data [28].

## Results

**Fig. 1:**
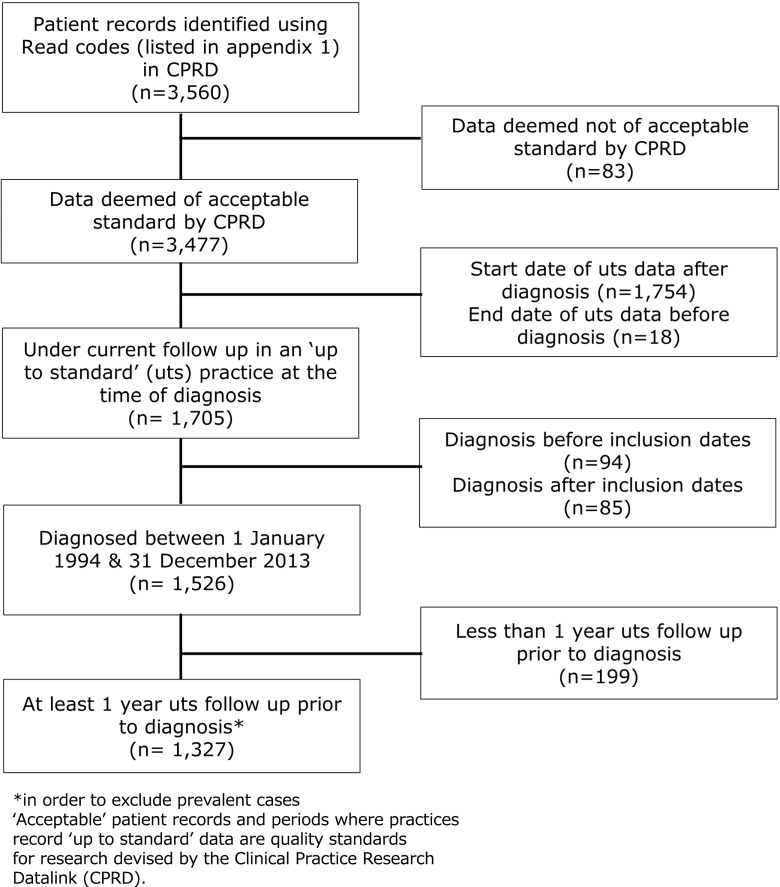
Flow diagram of ascertainment of incident cases of systemic sclerosis. Cases of systemic sclerosis ascertained from the Clinical Practice Research Datalink 1994–2013

Overall, we identified 1327 cases of systemic sclerosis in the CPRD during the study period (Fig. 1). Of these, 83.2% were female and the mean age at diagnosis was 58.0 years (SD 16.4).

### Incidence

The estimated overall incidence of systemic sclerosis in the entire CPRD population was 19.4 per million person-years (95% confidence interval (CI) 18.3–20.4) (Table 1). The incidence in adults aged ≥ 16 was 22.8 (95% CI 21.6–24.1) per million person-years and in children aged < 16 years was 2.9 (95% CI 2.1–4.1) per million person-years (Table 2).

**Table 1:** Annual incidence of systemic sclerosis 1994–2013 per million person-years in the Clinical Datalink

Year	Incident cases	Person- years	Incidence rate (95% CI)
1994	32	1,304,220	24.5 (17.4–34.7)
1995	36	1,406,927	25.6 (18.5–35.5)
1996	31	1,596,103	19.4 (13.7–27.6)
1997	30	1,884,001	15.9 (11.1–22.8)
1998	24	2,123,764	11.3 (7.6–16.9)
1999	47	2,576,123	18.2 (13.7–24.3)
2000	47	3,150,278	14.9 (11.2–19.9)
2001	58	3,459,183	16.8 (13.0–21.7)
2002	81	3,815,730	21.2 (17.1–26.4)
2003	79	4,039,225	19.6 (15.7–24.4)
2004	76	4,236,700	17.9 (14.3–22.5)
2005	92	4,352,147	21.1 (17.2–25.9)
2006	86	4,396,878	19.6 (15.8–24.2)
2007	94	4,434,807	21.2 (17.3–25.9)
2008	77	4,441,042	17.3 (13.9–21.7)
2009	82	4,451,968	18.4 (14.8–22.9)
2010	82	4,386,173	18.7 (15.1–23.2)
2011	103	4,285,455	24.0 (19.8–29.2)
2012	96	4,210,630	22.8 (18.7–27.8)
2013	74	3,978,397	18.6 (14.8–23.4)

Overall incidence = 19.4 (95% CI 18.3–20.4) per million person-years (1327 cases in 68,529,750 person-years)

**Table 2:** Incidence of systemic sclerosis 1994–2013 (per million person-years)

	Cases	Person-years	Crude incidence rate (95% CI)	Crude rate ratios (95% CI)	Adjusted rate ratios (95% CI)	P value^a^
Overall	1327	68,529,194	19.4 (18.3–20.4)			
Sex						
Male	223	34,069,626	6.5 (5.7–7.5)	1	1	< 0.0001
Female	1104	34,459,569	32.0 (30.2–34.0)	4.9 (4.2–5.7)	4.7 (4.1–5.4)	
Age group (years)						
Age 0–15	35	11,955,198	2.9 (2.1–4.1)	0.5 (0.3–0.7)	0.5 (0.3–0.7)	P_trend_ < 0.0001
Age 16–39	132	20,729,227	6.4 (5.4–7.6)	1	1	
Age 40–54	338	15,329,346	22.0 (19.8–24.5)	3.5 (2.8–4.2)	3.4 (2.8–4.2)	
Age 55–69	513	11,887,368	43.2 (39.6–47.1)	6.7 (5.6–8.2)	6.7 (5.5–8.1)	
Age 70–84	283	7,100,416	39.9 (35.3–44.8)	6.3 (5.1–7.7)	5.7 (4.6–7.0)	
Age 85+	26	1,528,196	17.0 (11.6–25.0)	2.7 (1.8–4.1)	2.1 (1.4–3.1)	
Each additional calendar year					1.0 (1.0–1.0)	P_trend_ 0.24
IMD 2010						
Quintile 1—most deprived	196	9,911,807	19.8 (17.2–22.7)	1	1	P_trend_ = 0.63
Quintile 2	187	9,728,120	19.2 (16.7–22.2)	1.0 (0.8–1.2)	0.9 (0.8–1.2)	
Quintile 3	157	8,356,976	18.8 (16.1–22.0)	1.0 (0.8–1.2)	1.0 (0.8–1.2)	
Quintile 4	152	7,802,398	19.5 (16.6–22.8)	1.0 (0.8–1.2)	1.1 (0.9–1.3)	
Quintile 5—least deprived	120	6,304,656	19.0 (15.9–22.7)	1.0 (0.8–1.2)	1.1 (0.9–1.4)	
IMD not known	515	26,425,793	19.5 (17.9–21.2)	1.0 (0.8–1.2)	1.0 (0.9–1.2)	

Crude incidence rate is calculated using univariable Poisson regression, and the adjusted rate ratio is calculated using multi-variable Poisson regression including sex, age-group, year of diagnosis and IMD-quintile as a priori confounders

^a^From multi-variable (adjusted) analysis using the likelihood ratio test

### Temporal trend

The incidence of systemic sclerosis did not change significantly between 1994 and 2013 (Table 1), adjusted rate ratio 0.6% increase per year (*P* = 0.24).

### Age, sex, socioeconomic status

The crude and adjusted incidence rate ratios are listed in Table 2. Incidence was higher among women than in men with an adjusted incidence rate ratio of 4.7 (95% CI 4.1–5.4), *P* < 0.0001. People aged 55–69 years had the highest incidence of systemic sclerosis. Socioeconomic status was not associated with incidence of systemic sclerosis, P_trend_ 0.63.

### Prevalence

Overall point prevalence in 2013 was 307 (95% CI 290–323) per million (Table 3). Prevalence was higher in women (adjusted OR 4.6, 95% CI 3.9–5.2, *P* < 0.0001) and highest in the 70–84 years category (835 per million, 95% CI 751–925), followed by the 55–69 years category (669 per million, 95% CI 612–730). There was no association between socioeconomic status and prevalence of systemic sclerosis, P_trend_ 0.68.

**Table 3:** Point prevalence of systemic sclerosis in 2013 (per million people)

	Cases	Denominator	Crude prevalence	Crude odds ratios (95% CI)	Adjusted Odds ratios (95% CI)	*P* value^1^
Overall	1337	4,362,809	307 (290–323)			
Sex						
Male	227	2,155,960	105 (92–120)	1	1	P < 0.0001
Female	1110	2,206,814	503 (474–533)	4.8 (4.1–5.5)	4.6 (3.9–5.2)	
Age group (years)						
Age 0–15	13	781,568	17 (9–28)	0.2 (0.1–0.4)	0.2 (0.1–0.4)	P_trend_ < 0.0001
Age 16–39	111	1,322,291	84 (69–101)	1	1	
Age 40–54	283	959,587	295 (262–331)	3.5 (2.8–4.4)	3.5 (2.8–4.4)	
Age 55–69	505	754,643	669 (612–730)	8.0 (6.5–9.8)	7.9 (6.5–9.8)	
Age 70–84	362	433,661	835 (751–925)	10.0 (8.0–12.3)	9.4 (7.6–11.7)	
Age 85+	63	111,059	567 (436–726)	6.8 (5.0–9.2)	5.6 (4.1–7.7)	
IMD 2010						
Quintile 1	188	600,110	313 (270–361)	1	1	P_trend_ = 0.68
Quintile 2	188	598,616	314 (271–362)	1.0 (0.8–1.2)	1.0 (0.8–1.2)	
Quintile 3	147	519,240	283 (239–333)	0.9 (0.7–1.1)	0.9 (0.8–1.2)	
Quintile 4	145	520,410	279 (235–328)	0.9 (0.7–1.1)	1.0 (0.8–1.3)	
Quintile 5	110	424,476	259 (213–312)	0.8 (0.7–1.0)	1.1 (0.8–1.3)	
IMD not known	559	1,699,957	329 (302–357)	1.0 (0.9–1.2)	1.1 (0.9–1.3)	

Crude odds ratio is calculated using univariable logistic regression, and adjusted odds ratio is calculated using multi-variable logistic regression including sex, age group, and IMD-quintile as a priori confounders

^a^From multi-variable (adjusted) analysis using the likelihood ratio test

### Survival

Over the study period, there were 302 deaths during 6929 years at risk contributed by people with incident systemic sclerosis generating a mortality rate of 43.6 (95% CI 38.9–48.8) per thousand person-years (Table 4). Risk factors for increased mortality were male sex and increasing age. However, there was no association between socioeconomic deprivation and mortality. The 1-, 5-, and 10-year survival was 94.2, 80.0, and 65.7% respectively.

**Table 4:** Mortality of systemic sclerosis 1994–2013 (per thousand person-years)

	Cases	Deaths	Years at risk	Crude mortality	Crude hazard ratios (95% CI)	Adjusted hazard ratios (95% CI)	*P* value^a^
Overall	1327	302	6929	43.6 (38.9–48.8)			
Sex							
Male	223	53	1119	47.4 (36.2–62.0)	1	1	P = 0.06
Female	1104	249	5810	42.9 (37.9–48.5)	0.9 (0.7–1.2)	0.7 (0.5–1.0)	
Age group (years)							
Age 0–15	35	1	136	7.4 (1.0–52.2)	1.2 (0.1–11.9)	1.2 (0.1–11.4)	P_trend_ < 0.0001
Age 16–39	146	3	501	6.0 (1.9–18.6)	1	1	
Age 40–54	390	20	1572	12.7 (8.2–19.7)	2.2 (0.6–7.3)	1.2 (0.7–7.6)	
Age 55–69	668	89	2923	30.4 (24.7–37.5)	5.4 (1.7–17.0)	5.7 (1.8–17.9)	
Age 70–84	473	152	1616	94.1 (80.3–110.3)	16.7 (5.3–52.3)	17.7 (5.6–55.7)	
Age 85+	75	37	182	203.7 (147.6–281.2)	36.2 (11.1–117.8)	37.8 (11.6–123.2)	
IMD 2010^b^							
Quintile 1	196	45	1048	42.9 (32.1–57.5)	1	1	P_trend_ = 0.49
Quintile 2	187	42	999	42.1 (31.1–56.9)	1.0 (0.6–1.5)	1.0 (0.7–1.6)	
Quintile 3	157	36	702	51.3 (37.0–71.1)	1.2 (0.8–1.8)	1.0 (0.6–1.5)	
Quintile 4	152	37	795	46.6 (33.7–64.3)	1.1 (0.7–1.7)	1.0 (0.7–1.6)	
Quintile 5	120	37	628	58.9 (42.7–81.3)	1.3 (0.9–2.1)	1.2 (0.8–1.9)	
IMD not known	515	105	2758	38.1 (31.4–46.1)	0.9 (0.6–1.3)	0.9 (0.6–1.2)	

Crude hazard ratio is calculated using univariable Cox regression, and adjusted hazard ratio is calculated using multi-variable Cox regression including sex, age group, and IMD-quintile as a priori confounders

^a^From multi-variable (adjusted) analysis, using the likelihood ratio test

^b^Quintile 1 is the most deprived, Quintile 5 is the least deprived

### Estimated UK incidence and prevalence

We estimate that at present, there are 1180 new cases of systemic sclerosis each year in the UK and 19,390 people living with systemic sclerosis. Population projections show a large expected increase in the proportion of the UK population in the 55+ age group [29] in 20 years’ time, meaning that we estimate in 2037, there will be 1460 new cases (24% increase) and 24,430 people living with systemic sclerosis (26% increase).

## Discussion

### Main findings

Using the CPRD, we estimate that the annual incidence of systemic sclerosis in the UK population is 19.4 per million person-years. The incidence is nearly five times higher in women than in men, is not influenced by socioeconomic status, and has remained stable over the 20-year study period. The peak age of onset is 55–69 years. We estimate the UK prevalence of systemic sclerosis in the UK population is 307 (290–323) per million with the highest prevalence in the 70–84 years age group.

### How our study fits in with other literature

Our study estimated incidence and prevalence in the UK to be higher than previously reported in Norway, Croatia, Greece, France, Taiwan, and Australia [6, 7, 10, 12, 13, 16], similar to the USA, Sweden, and Spain [11, 14, 15], and lower than in Italy and Canada [3, 4]. Our findings were consistent with other large population-based studies in Caucasians. A large study in the Detroit tri-county area of the USA between 1989 and 1991 estimated the incidence of systemic sclerosis to be 19.3 per million but with a lower prevalence of 242 per million, using 5 data sources including hospital records and a capture-recapture analysis [15]. A more recent study conducted in southern Sweden used a health register of 1.2 million pooled public and private health care records between 2006 and 2010 and estimated an incidence of 19 per million and a prevalence of 305 per million [14]. This study had approval to retrospectively review the medical records and validate the diagnosis of systemic sclerosis with reference to the 1980 ARA criteria [30], and the similarity between our findings support the reliability of our case ascertainment. Taken together [14], our studies challenge the idea that the prevalence of systemic sclerosis is lower in Europe than in the USA and Australia [8], or that it is lower in Northern Europe compared to Southern Europe [8].

Our estimates of incidence and prevalence are much higher than previous UK estimates. Our study is the first nationwide UK study of incidence and prevalence of systemic sclerosis, and the first of a prospectively collected healthcare database. Our estimate of incidence among adults is more than 4 times higher than the previous estimates of 4 per million from the west Midlands in the 1980s [1], and our estimate of incidence among children was 10 times higher than the previous estimate of 0.27/million across the whole UK in 2010 [31]. Our estimate of prevalence is 3.5 times higher than the most recent estimate of 8.4 per million from North East England in 2004 [5]. The lower incidence and prevalence reported previously are likely to be methodological, caused by under-estimation of cases in these studies due to their reliance on physicians (nationally or in a limited geographical area) being asked to record cases of systemic sclerosis attending their clinics and reporting this to the authors [1, 31], or on physician recall [5] which are not as reliable as prospectively recorded diagnoses in the patient’s general practice record.

The higher incidence and prevalence among women than men is well-recognized. Ours is the first study to report the effect of socioeconomic status on the incidence of systemic sclerosis; we observed no effect of socioeconomic status, as estimated by area-based deprivation data which is used as a proxy for socioeconomic status and social class. This lack of effect is in contrast to other autoimmune diseases such as rheumatoid arthritis and systemic lupus erythematosus where increasing deprivation is associated with increased incidence and disease severity [32–34]. Survival at 1, 5, and 10 years of 94.2, 80.0, and 65.7% respectively is very similar to the pooled estimates from an international meta-analysis of mortality in systemic sclerosis using individual patient data [35]. Mortality was increased in older people, and possibly men compared to women, but unaffected by socioeconomic status.

The mean age of diagnosis in our study was 58 years (SD 16.4 years). While this is consistent with a previous UK study [5] and a study using inpatient and outpatient healthcare databases in Northern Italy [3], it is higher than the established teaching that onset is greatest from early adulthood to the 4th decade [36] and higher than most recent studies which report the mean age of onset as 45–48 years [1, 6, 7]. It is unclear why this is. It could be that there are longer delays between symptoms onset and diagnosis in our healthcare system than in others. There is also a possible delay after the diagnosis and before it is recorded in the CPRD. Such delay has recently been quantified in rheumatoid arthritis in the CPRD and was found to be < 2 years [37].

Systemic sclerosis is an important condition for rheumatologists, because although rare, it has a very high mortality compared to other musculoskeletal diseases [38], and optimal patient care is challenging and involves multidisciplinary effort. We have found a much higher incidence and prevalence of systemic sclerosis in the UK than previously reported [38] and in addition have estimated that the burden on our healthcare services will increase by 25% over the next 20 years as a result of changes in population demographics.

### Strengths

This study is the first nationwide European study and largest of its kind in Europe. The main strength of our study is the use of the CPRD dataset. It is the largest primary care database, containing more than 13 million patient records and covering approximately 6% of the UK population. It has been validated as a representative dataset so results can be applied to the UK population as a whole [20]. It has good quality demographic data, allowing us to study the influence of age, sex, and socioeconomic status on incidence, prevalence, and survival of systemic sclerosis. It has allowed us to conduct a population-based study in a prospectively collected dataset, which avoids the selection bias of cohort studies from tertiary referral centers and recall bias of studies relying on physician memory.

### Limitations

Systemic sclerosis is a rare disease, and the number of cases identified is small in comparison with some other conditions. Despite this, we have identified a cohort of more than 1300 people with systemic sclerosis which allows us to make the most precise estimates of incidence, prevalence, and survival published in a European population, which are essential for health service planning.

Within the CPRD, it is not possible to externally validate the date of diagnosis and accuracy of diagnosis of systemic sclerosis. In the past, it was possible to request anonymized sets of hospital correspondence, but this service has been withdrawn by the CPRD to increase the confidentiality of the database. Previous studies of the accuracy of the recording of diagnosis of similar chronic diseases in the CPRD have shown positive predictive values (PPVs) > 90% [39]. For example, the PPV of codes for granulomatosis with polyangiitis (formerly known as Wegener’s granulomatosis) and idiopathic thrombocytopenic purpura were both found to be 91% [23, 24]. There is no reason to believe it should be dissimilar in systemic sclerosis because a diagnosis of all of these serious autoimmune diseases are very unlikely to be recorded by a GP without confirmation from secondary care, where the diagnosis would have been made [25, 40]. It is therefore also not possible to apply classification criteria, but our study represents people considered to have systemic sclerosis by their physicians. It is also unknown how many people may be undiagnosed, which means that our estimates, like all others, are likely to be underestimates of incidence and prevalence.

The Read codes do not allow cases to be differentiated into diffuse cutaneous and limited cutaneous phenotypes of systemic disease; however, we have excluded morphoea and other localized forms of cutaneous only disease. Our study therefore contains people with systemic sclerosis but cannot comment on differences between diffuse and limited cutaneous phenotypes.

### Conclusion

We found the UK incidence and prevalence of systemic sclerosis to be higher than previously reported in the UK but similar to other recent USA and European estimates [14, 15]. Our findings suggest that systemic sclerosis is not less common in Europe than in the USA and Australia and not less common in northern Europe compared to southern Europe.

## References

[1] Silman AJ, Jannini S, Symmons DPM (1988). An epidemiological study of scleroderma in the west midlands. Br J Rheumatol.

[2] Kaipiainen-Seppanen O, Aho K (1996). Incidence of rare systemic rheumatic and connective tissue diseases in Finland. J Intern Med.

[3] Lo Monaco A, Bruschi M, La Corte R (2011). Epidemiology of systemic sclerosis in a district of northern Italy. Clin Exp Rheumatol.

[4] Bernatsky S, Joseph L, Pineau CA, Belisle P, Hudson M, Clarke AE (2009). Scleroderma prevalence: demographic variations in a population-based sample. Arthritis Care Res.

[5] Allcock RJ, Forrest I, Corris PA, Crook PR, Griffiths ID (2004). A study of the prevalence of systemic sclerosis in Northeast England. Rheumatology (Oxford).

[6] Kuo C-F, See L-C, Yu KH, Chou IJ, Tseng WY, Chang HC, Shen YM, Luo SF (2011). Epidemiology and mortality of systemic sclerosis: a nationwide population study in Taiwan. Scand J Rheumatol.

[7] Le Guern V, Mahr A, Mouthon L (2004). Prevalence of systemic sclerosis in a French multi-ethnic county. Rheumatology.

[8] Chifflot H, Fautrel B, Sordet C, Chatelus E, Sibilia J (2008). Incidence and prevalence of systemic sclerosis: a systematic literature review. Semin Arthritis Rheum.

[9] Geirsson AJ, Steinsson K, Gudmundsson S (1994). Systemic sclerosis in Iceland. A nationwide epidemiological study. Ann Rheum Dis.

[10] Alamanos Y, Tsifetaki N, Voulgari PV (2005). Epidemiology of systemic sclerosis in Northwest Greece 1981 to 2002. Semin Arthritis Rheum.

[11] Arias-Nuñez MC, Llorca J, Vazquez-Rodriguez TR, Gomez-Acebo I, Miranda-Filloy JA, Martin J, Gonzalez-Juanatey C, Gonzalez-Gay MA (2008). Systemic sclerosis in northwestern Spain: a 19-year epidemiologic study. Medicine (Baltimore).

[12] Radić M, Kaliterna DM, Fabijanić D, Radić J (2010). Prevalence of systemic sclerosis in Split-Dalmatia county in southern Croatia. Clin Rheumatol.

[13] Hoffmann-Vold AM, Midtvedt Ø, Molberg Ø, Garen T, Gran JT (2012). Prevalence of systemic sclerosis in south-east Norway. Rheumatol (United Kingdom).

[14] Andreasson K, Saxne T, Bergknut C (2014). Prevalence and incidence of systemic sclerosis in southern Sweden: population-based data with case ascertainment using the 1980 ARA criteria and the proposed ACR-EULAR classification criteria. Ann Rheum Dis.

[15] Mayes MD, Lacey JV, Beebe-Dimmer J, Gillespie BW, Cooper B, Laing TJ, Schottenfeld D (2003). Prevalence, incidence, survival, and disease characteristics of systemic sclerosis in a large US population. Arthritis Rheum.

[16] Roberts-Thomson PJ, Walker JG, Lu TYT, Esterman A, Hakendorf P, Smith MD, Ahern MJ (2006). Scleroderma in South Australia: further epidemiological observations supporting a stochastic explanation. Intern Med J.

[17] Silman a J, Howard Y, Hicklin AJ (1990). Geographical clustering of scleroderma in south and west London. Br J Rheumatol.

[18] Walley T, Mantgani A (1997). The UK general practice database. Lancet.

[19] Office of National Statistics, 2011 Census [Internet]

[20] Herrett E, Gallagher AM, Bhaskaran K, Forbes H, Mathur R, van Staa T, Smeeth L (2015). Data resource profile: Clinical Practice Research Datalink (CPRD). Int J Epidemiol.

[21] Dave S, Petersen I (2009). Creating medical and drug code lists to identify cases in primary care databases. Pharmacoepidemiol Drug Saf.

[22] Springate DA, Kontopantelis E, Ashcroft DM, Olier I, Parisi R, Chamapiwa E, Reeves D (2014). ClinicalCodes: an online clinical codes repository to improve the validity and reproducibility of research using electronic medical records. PLoS One.

[23] Watts RA, Al-Taiar A, Scott DGI (2009). Prevalence and incidence of Wegener’s granulomatosis in the UK general practice research database. Arthritis Rheum.

[24] Marieke Schoonen W, Kucera G, Coalson J, Li L, Rutstein M, Mowat F, Fryzek J, Kaye JA (2009). Epidemiology of immune thrombocytopenic purpura in the general practice research database. Br J Haematol.

[25] Rees F, Doherty M, Grainge M et al (2015) The incidence and prevalence of systemic lupus erythematosus in the UK, 1999-2012. Ann Rheum Dis Published Online First: September 2014. 10.1136/annrheumdis-2014-206334 10.1136/annrheumdis-2014-206334 PMC4717400 25265938

[26] Lewis JD, Bilker WB, Weinstein RB, Strom BL (2005). The relationship between time since registration and measured incidence rates in the general practice research database. Pharmacoepidemiol Saf.

[27] CPRD linked data. https://www.cprd.com/dataAccess/linkeddata.asp#Deprivationdata (accessed 24 Apr 2017)

[28] Benchimol EI, Smeeth L, Guttmann A, Harron K, Moher D, Petersen I, Sørensen HT, von Elm E, Langan SM, RECORD Working Committee (2015). The REporting of studies conducted using observational routinely-collected health data (RECORD) statement. PLoS Med.

[29] Office for National Statistics (2015) Population Estimates for UK, England and Wales, Scotland and Northern Ireland. https://www.ons.gov.uk/peoplepopulationandcommunity/populationandmigration/populationestimates/datasets/populationestimatesforukenglandandwalesscotlandandnorthernireland (accessed 18 Oct 2017)

[30] Masi AT (1980). Preliminary criteria for the classification of systemic sclerosis (scleroderma). Arthritis Rheum.

[31] Herrick AL, Ennis H, Bhushan M, Silman AJ, Baildam EM (2010). Incidence of childhood linear scleroderma and systemic sclerosis in the UK and Ireland. Arthritis Care Res (Hoboken).

[32] Bengtsson C (2005). Socioeconomic status and the risk of developing rheumatoid arthritis: results from the Swedish EIRA study. Ann Rheum Dis.

[33] Pedersen M, Jacobsen S, Klarlund M, Frisch M (2006). Socioeconomic status and risk of rheumatoid arthritis: a Danish case-control study. J Rheumatol.

[34] Trupin L, Tonner MC, Yazdany J, Julian LJ, Criswell LA, Katz PP, Yelin E (2008). The role of neighborhood and individual socioeconomic status in outcomes of systemic lupus erythematosus. J Rheumatol.

[35] Ioannidis JPA, Vlachoyiannopoulos PG, Haidich A-B, Medsger TA, Lucas M, Michet CJ, Kuwana M, Yasuoka H, van den Hoogen F, te Boome L, van Laar JM, Verbeet NL, Matucci-Cerinic M, Georgountzos A, Moutsopoulos HM (2005). Mortality in systemic sclerosis: an international meta-analysis of individual patient data. Am J Med.

[36] Denton CP, Moinzadeh P (2013) Systemic sclerosis. In: Watts RA, Conaghan PG, Denton C et al (eds) Oxford textbook of rheumatology. doi: 10.1093/med/9780199642489.003.0121

[37] Ford E, Carroll J, Smith H, Davies K, Koeling R, Petersen I, Rait G, Cassell J (2016). What evidence is there for a delay in diagnostic coding of RA in UK general practice records? An observational study of free text. BMJ Open.

[38] Parsons S, Ingram M, Clarke-Cornwell AM et al (2011) A heavy burden: the occurrence and impact of musculoskeletal conditions in the United Kingdom today 41. https://www.escholar.manchester.ac.uk/uk-ac-man-scw:123774

[39] Herrett E, Thomas SL, Schoonen WM, Smeeth L, Hall AJ (2010). Validation and validity of diagnoses in the general practice research database: a systematic review. Br J Clin Pharmacol.

[40] Dalleywater W, Powell HA, Hubbard RB, Navaratnam V (2015). Risk factors for cardiovascular disease in people with idiopathic pulmonary fibrosis: a population-based study. Chest.

